# Interleukin-6-174G>C gene promoter polymorphism and prognosis in patients with cancer

**DOI:** 10.18632/oncotarget.17771

**Published:** 2017-05-10

**Authors:** Kan Zhai, Yong Yang, Zhi-Gang Gao, Jie Ding

**Affiliations:** ^1^ Medical Research Center, Beijing Chao-Yang Hospital, Capital Medical University, Beijing 100020, China; ^2^ Department of General Surgery, Beijing Chao-Yang Hospital, Capital Medical University, Beijing 100020, China

**Keywords:** Interleukin-6, polymorphism, cancer, prognosis, meta-analysis

## Abstract

Interleukin-6 (IL-6) is known to be involved in the pathogenesis of cancer progression. IL-6-174G>C polymorphism has shown several results in association studies. In this study, we evaluated the association the IL-6-174G>C polymorphism and overall survival (OS) of cancer using 17 eligible studies with 4,304 patients. Our meta-analysis indicated that IL-6-174G>C polymorphism is not associated with OS when assessed using 3 genotype comparison including GG/(GC+CC), CC/(GC+GG) and CC/GG. Interestingly, compared to GG carrier, patients with IL-6-174GC genotype showed a decreased hazard of poor OS (hazard ratio = 0.81, 95% confidence interval: 0.68–0.96, *P* = 0.018; I^2^ = 34.5%, *P*het = 0.107). However, for GG/(GC+CC) genotype comparison, this SNP is affect patients’ OS obviously in bladder cancer, ovarian and peritoneal cancer, neuroblastoma, gastric cancer and osteosarcoma, though pooled results showing negative association because adverse and protective effect on different type of cancer balance each other. These results suggest IL-6-174G>C polymorphism might play a role in modulating OS in different type of cancer and might contribute to individual treatment in the future.

## INTRODUCTION

The prognosis of cancer is mainly affected by the cancer type, histological characteristics, tumor stage and therapy. Early diagnosis and accurate prognosis prediction is very necessary for cancer patients. Inflammation has a strong association with cancer progression because malignant cells are promoted by the inflammatory cytokines in a microenvironment are to be highly proliferative [1].

In 1985, interleukin-6 (IL-6) was first discovered as a B cell differentiation factor inducing immature B cells to produce antibody [2]. This cytokine is a general marker of inflammation, which plays pro-inflammatory and anti-inflammatory mediator for its regulating immune response, inflammation and different pathophysiologic process [3–5]. IL-6 is expressed in a diverse range of cell types, for example, B cells, T cells, fibroblasts, macrophages and adipose cells [6]. Several studies show that evaluated IL-6 is associated with many type of tumors such as breast carcinoma, colorectal cancer, lung cancer, ovarian cancer and lymphomas [7–11]. IL-6 could facilitate carcinogenesis through several mechanisms, including apoptosis, survival, proliferation, angiogenesis, invasiveness, metastasis and metabolism [12].

Fishman et al. [13] first reported a single nucleotide polymorphism (SNP) at the promoter region of the IL-6 in 1998. This functional SNP (rs1800795) which is located in -174 is related to the constitutively IL-6 transcription rate, which could control the levels of IL-6. Compared to -174CC genotype carriers, -174GG/GC genotype carriers have a higher IL-6 expression [13]. Accumulating evidence has suggested IL-6-174G>C is associated with susceptibility to many types of cancer [14]. Published studies also suggest that this SNP is associated with the prognosis of cancer, including non-small cell lung cancer, bladder cancer, neuroblastoma, breast cancer [15–19]. Although the IL-6-174G>C polymorphism has been assessed in association with survival of several types of cancer, the results are still inconsistent.

In the present study, we performed the first comprehensive meta-analysis of available studies to obtain a comprehensive evaluation of the association between IL-6-174 polymorphism and cancer prognosis.

## RESULTS

### Eligible Studies

The initial search retrieved 78 studies using different combinations of key words. Fourth-eight studies remained and were obtained in full-text review after deleting duplication. Among them, 4 were excluded for review or other studies except human cancer. Twenty-four were excluded because of no data about IL-174G>C and OS. Two was excluded for retraction. One study [20] was also excluded for plagiarizing others’ work [19]. With strict inclusion and exclusion criteria, the primary eligible studies including 17 studies involving a total of 4,304 cancer patients. Table 1 shows the general characteristics of eligible studies. Fifteen studies were conducted on Caucasian [15, 16, 18, 19, 21–31], 1 were on American [32], and 1 was on Asian [33]. Polymerase chain reaction-restriction fragment length polymorphism (PCR-RFLP), PCR-Sequencing, single-strand conformation polymorphism (SSCP), polymerase chain reaction-sequencing (PCR- sequencing), TaqMan and Sequenom were used.

**Table 1:** Characteristics of eligible studies in this meta-analysis

Study	No. of cases	Country	Ethnicity	Cancer type	Genotyping method
DeMichele et al. (2003)	124	America	Caucasian	Breast cancer	PCR-RFLP
Hefler et al. (2003)	120	Germany	Caucasian	Ovarian cancer	PCR-Sequencing
Iacopetta et al. (2004)	256	Australia	Caucasian	Breast cancer	PCR-SSCP
Leibovici et al. (2005)	149	America	Caucasian	Bladder cancer	TaqMan
Garg et al. (2006)	160	America	Caucasian	Ovarian and peritoneal cancer	PCR-Sequencing
Deans et al. (2007)	203	United Kingdom	Caucasian	Gastric or oesophageal cancer	TaqMan
Wilkening et al. (2008)	303	Sweden	Caucasian	Colorectal cancer	TaqMan
Cherel et al. (2009)	293	France	Caucasian	Breast cancer	PCR-RFLP
DeMichele et al. (2009)	328	America	Caucasian	Breast cancer	PCR-Sequencing
Lagmay et al. (2009)	96	America	Caucasian	Neuroblastoma	PCR-RFLP
Lopez et al. (2011)	445	Brasil	American	Head and neck cancer	TaqMan
Totaro et al. (2013)	326	Italy	Caucasian	Neuroblastoma	PCR-RFLP
Markkula et al. (2014)	574	Sweden	Caucasian	Breast cancer	Sequenom
Ruzzo et al. (2014)	161	Italy	Caucasian	Gastric cancer	PCR-RFLP
Gomes et al. (2015)	434	Portugal	Caucasian	Non-small cell lung cancer	PCR-RFLP
Matsusaka et al. (2016)	187	Italy	Caucasian	Colorectal cancer	PCR-Sequencing
Qi et al. (2016)	216	China	Asian	Osteosarcoma	PCR-RFLP

Abbreviations: PCR-RFLP, polymerase chain reaction-restriction fragment length polymorphism; PCR-SSCP, single-strand conformation polymorphism

### Overall survival of IL-6-174G>C polymorphism

**Figure 1:**
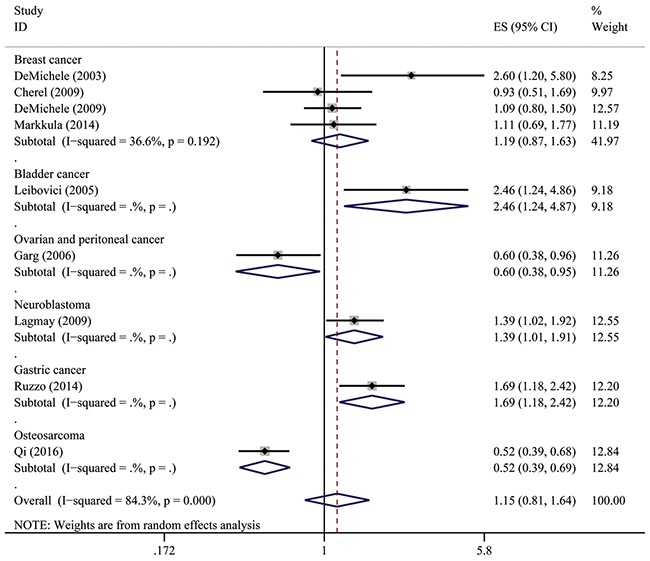
Overall association between IL-6-174G>C polymorphism and OS of cancer (GG/GC+CC) For each study, the estimate of HR and its 95% (CI) is plotted with a box and a horizontal line. The symbol diamond indicates pooled HR and its 95% CI.

**Figure 2:**
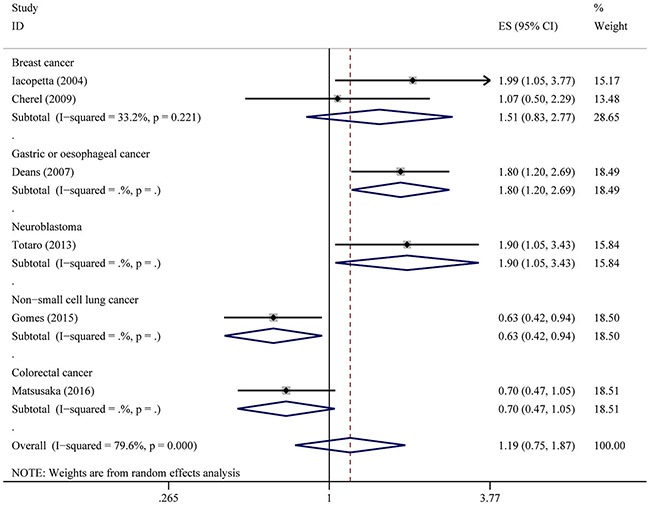
Overall association between IL-6-174G>C polymorphism and OS of cancer (CC/GC+GG) For each study, the estimate of HR and its 95% (CI) is plotted with a box and a horizontal line. The symbol diamond indicates pooled HR and its 95% CI.

**Figure 3:**
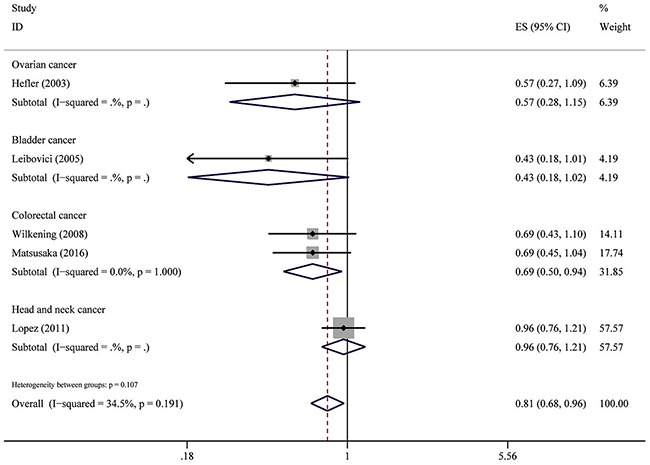
Overall association between IL-6-174G>C polymorphism and OS of cancer (GC/GG) For each study, the estimate of HR and its 95% (CI) is plotted with a box and a horizontal line. The symbol diamond indicates pooled HR and its 95% CI.

The associations between IL-6-174G>C polymorphism and OS of cancer in each eligible studies are shown in Table 2. Because of data quality, we assessed data using several genetic models. Of the combined 9 studies with 2,057 patients, results show that IL6-174GG genotype was not associated with OS compared to CC and CG genotype (HR = 1.15, 95% CI: 0.81–1.64, *P* = 0.436; I^2^ = 84.3%, *P*het < 0.001). However, forest plot shows that this SNP is associated with OS with adverse or protective effect on each group, except for breast cancer group (Figure 1). Similar results were also observed in IL6-174CC genotype compared to GC and GG genotype including 6 studies with 1,681 patients (HR = 1.19, 95% CI: 0.75–1.87, *P* = 0.458; I^2^ = 79.6%, *P*het = 0.458) (Figure 2). Five studies comprising of 1,195 patients was analyzed using genotype model GC/GG for an association between IL-6-174 polymorphism and OS. The pooled HR was statistically significant (HR = 0.81, 95% CI: 0.68–0.96, *P* = 0.018; I^2^ = 34.5%, *P*het = 0.107) (Figure 3).

**Table 2:** Summary of primary data for association between IL-6-174G>C polymorphism and overall survival of cancer from 17 eligible studies

Study	HR (95% CI)			
	GG/(GC+CC)	CC/(GC+GG)	GC/GG	CC/GG
DeMichele et al. (2003)	2.60 (1.20–5.80)			
Hefler et al. (2003)			0.57 (0.27–1.09)	1.58 (0.37–6.72)
Iacopetta et al. (2004)		1.99 (1.05–3.77)		
Leibovici et al. (2005)	2.46 (1.24–4.86)		0.43 (0.18–1.01)	0.42 (0.13–1.34)
Garg et al. (2006)	0.60 (0.38–0.96)			
Deans et al. (2007)		1.80 (1.20–2.69)		
Wilkening et al. (2008)			0.69 (0.43–1.10)	1.55 (0.88–2.72)
Cherel et al. (2009)	0.93 (0.51–1.69)	1.07 (0.50–2.29)		
DeMichele et al. (2009)	1.09 (0.80–1.50)			
Lagmay et al. (2009)	1.39 (1.02–1.92)			
Lopez et al. (2011)			0.96 (0.76–1.21)	1.13 (0.78–1.64)
Totaro et al. (2013)		1.90 (1.05–3.43)		
Markkula et al. (2014)	1.11 (0.69–1.77)			
Ruzzo et al. (2014)	1.69 (1.18–2.42)			
Gomes et al. (2015)		0.63 (0.42–0.94)		
Matsusaka et al. (2016)		0.70 (0.47–1.05)	0.69 (0.45–1.04)	0.85 (0.38–1.87)
Qi et al. (2016)	0.52 (0.39–0.68)			

**Figure 4:**
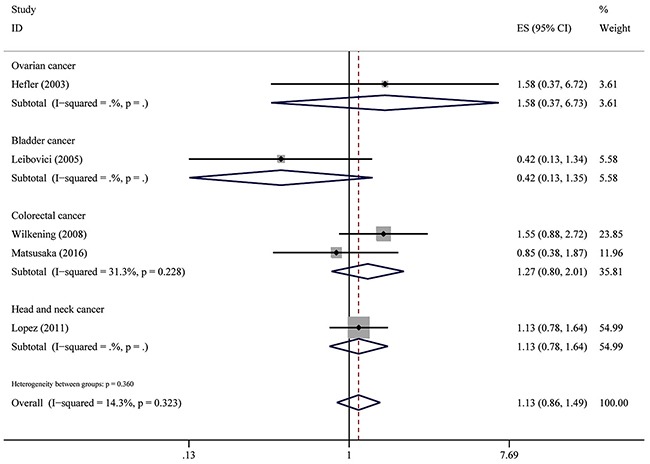
Overall association between IL-6-174G>C polymorphism and OS of cancer (CC/GG) For each study, the estimate of HR and its 95% (CI) is plotted with a box and a horizontal line. The symbol diamond indicates pooled HR and its 95% CI.

For CC/GG genotype model, no association was found with a total of studies and patients. (HR = 1.13, 95% CI: 0.86–1.49, *P* = 0.392; I^2^ = 14.3%, *P*het = 0.360) (Figure 4).

### Sensitivity analysis and publication bias

To evaluate the influence of every study on the pooled HRs in each genetic model, we performed sensitivity analysis. The results indicated that excluding each study did not influence the overall effects, suggesting that this meta-analysis were reliable.

Begg's and Egger's test were performed to evaluate the publication bias of all included studies in each genetic model. The results indicate that no evidence of obvious asymmetry in overall analysis of publication bias for IL-6-174G>C polymorphism in each genetic model (data not shown).

## DISCUSSION

Functional genetic polymorphisms such as SNPs in the promoter potentially affecting expressions of certain genes might be important determinants in malignancies. Accumulating evidences suggest SNPs in IL-6 which is crucial cytokine involved in several cellular pathways may affect survival of cancer. However, the association remains uncertain. Therefore, we performed the first meta-analysis to evaluate IL-6-174G>C in predicting clinical outcomes of cancer. Our results indicate that there might be a connection between IL-6 polymorphism and OS in patients with cancer. Our study also highlights the effect of constitutively higher IL-6 expression in cancer prognosis.

IL-6 mediates its roles through IL-6 receptor on the plasma membrane, and this complex along with gp130 and lead to form an activated IL-6 complex [34, 35]. This complex exerts its effects activation of several pathways, including MAP kinase, JAK/STAT, PI3K/AKT and NF-B pathways, leading to the transcriptional regulation of key genes in cell survival, proliferation, differentiation, metabolism, angiogenesis, inflammation, invasion, and metastasis [12].

Published studies suggest that evaluated expression of IL-6 is associated with cancer risk at the time of diagnosis. These results do not contradict that IL-6-174C inducing higher constitutively IL-6 expression associated with increase or decrease survival. Considering heterogeneous malignancies, it's suggested that the level of IL-6 expression might play a different important role at the step of each cancer progression, although the reasons for this have not been understood. For example, studies indicate that IL-6-174G>C are correlated with altered breast cancer survival of different race and hormone status [36].

The main strength of this meta-analysis was its well defined search strategy and data extraction according to the strict inclusion and exclusion criteria. Because it's difficult to obtain data, there are few studies about IL-6 polymorphism and cancer prognosis. Several genetic models are used in these studies, it's impossible to assess IL-6-174G>C using one genetic model in all included studies. In our study, we calculated the association with this SNP using 4 genotype models and OS of cancer patients. Although 3 of 4 pooled results showing no association with OS, it is affect patients survival evidently, because adverse and protective effect on different type of cancer balances each other. Lippitz and Harris published a review and reported the serum level of IL-6 was significantly associated with survival in 85.6% of cancer patients (9917/11,583) in 23 different types of cancer [37]. They pointed that the evaluated level of IL-6 in serum correlates with survival as paraneoplastic condition in later cancer stages independent of cancer type [37]. Considering IL-6-174G>C could affect the expression of IL-6 protein, independent of cancer type, clinical status of patients included in our meta-analysis could not be uniformed, therefore, we think this might be the reason of negative pooled association using the three genetic models.

There are several limitations in our meta-analysis. First, number of included studies and in this analysis is small. Patterns of genetic models using in published studies are varied. The statistic power of each genetic model was still limited because of the relatively small sample size. Besides genetic polymorphism, other factors, such as tumor microenvironment, therapy and their interaction could contribute to cancer prognosis. For example, in this study, compared to GC+CC genotype, GG carrier is associated with OS with adverse or protective effect on types of cancer including bladder cancer, ovarian and peritoneal cancer, neuroblastoma, gastric cancer and osteosarcoma, except for breast cancer. It's reported that IL-6 genotype is strongly associated with OS with hormone level [15, 29]. Because of lacking information, the present study did not combine the effects of these multiple factors in the analyses between IL-6 polymorphism and OS. Third, there are several SNPs in IL-6 which reported to be correlated to cancer risk or survival. However, -174G>C is the most famous SNP in IL-6 promoter region which is related to cancer. The present study only assessed -174G>C in the promoter, though it was not possible to analyze all SNPs.

In summary, our results suggest a role for IL6-174G>C polymorphism in cancer prognosis. Because of limited number of cases in the present study, further studies are needed to evaluate the significance of these results in each type of cancer in the clinic.

## MATERIALS AND METHODS

### Publication Search

We searched three electronic databases PubMed, Embase and Web of Science up January 2017 for all related studies reporting the genotype of IL-6-174 polymorphism (rs1800795) in cancer patients, using different combinations of the key words ‘IL-6 or IL6’, ‘-174’, ‘rs1800795’, ‘cancer’, and ‘survival’, without any restriction. The literature search was limited to studies that had been conducted on human subjects. The reference lists of the retrieved articles, reviews and editorials were also screened to find all additional eligible studies.

### Inclusion Criteria

The studies selected had to meet the following criteria: (1) all patients diagnosed with cancer should be histologically confirmed; (2) study evaluated of the IL-6-174 polymorphism in patients with cancer and overall survival (OS); (3) published in English; (4) subjects were limited to adult and without autoimmune diseases, studies with children were also excluded; (5) genotyping was based on DNA; (6) sufficient data for estimating hazard ratio (HR) and its corresponding 95% confidence interval (95% CI) for OS. When publications were checked for overlapping patients, only the largest or complete study was selected.

### Data extraction

An initial screening of title and abstract was performed in the first step, followed by further screening based on full-text review. Information was independently collected from all eligible studies by two investigators (K.Z. and Y.Y.), including the first author, publication date, country of the study location, ethnicity of the study population, number of genotyped cases, genotyping method, type of cancer, HR and its 95% CI for OS. For studies with insufficient information on HRs in primary reports, we extracted data from OS curves. Disagreements were resolved through discussion.

### Statistical analysis

HRs with corresponding 95% CIs for OS was obtained from each included studies. For studies which data were not directly recorded in primary reports, we calculated HR and their 95% CIs from survival curves using published method [38, 39]. Kaplan-Meier curves of included studies were read by Engauge Digitizer version 4.1. HR calculation was determined as described in previous published study [40]. Z test was used to determine the pooled HR and 95% CI. Heterogeneity among studies was examined with I^2^ statistics. I^2^ < 50% indicates no significant heterogeneity between studies, HRs were evaluated using fixed-effect model (Mantel-Haenszel method). Otherwise, random-effect model (DerSimonian-Laird method) was used. The potential publication bias was assessed using Begg's funnel plot and Egger's linear regression test [41, 42]. Sensitivity analyses by excluding every study and recalculating HRs and 95% CI were conducted [43]. All statistical tests were carried out with Stata 12.0 (StataCorp, College Station, TX, USA). A 2-tailed *P* < 0.05 was considered as statistical significance.
